# Single nucleotide polymorphisms in the *KRT82* promoter region modulate irregular thickening and patchiness in the dorsal skin of New Zealand rabbits

**DOI:** 10.1186/s12864-024-10370-7

**Published:** 2024-05-10

**Authors:** Bohao Zhao, Jiawei Cai, Xiyu Zhang, Jiali Li, Zhiyuan Bao, Yang Chen, Xinsheng Wu

**Affiliations:** 1 College of Animal Science and Technology, Yangzhou University, Yangzhou, 225009 Jiangsu China; 2 Joint International Research Laboratory of Agriculture & Agri-Product Safety, Yangzhou University, Yangzhou, 225009 Jiangsu China

**Keywords:** Rabbit, Patchiness phenotype, Skin, Hair follicle, *KRT82*, Single nucleotide polymorphism

## Abstract

**Background:**

While rabbits are used as models in skin irritation tests, the presence of irregular patches and thickening on the dorsal skin can affect precise evaluation. In this study, genes associated with patchiness or non-patchiness on the dorsal skin of New Zealand rabbits were investigated to identify potential regulators of the patchiness phenotype.

**Results:**

The results showed that parameters associated with hair follicles (HFs), such as HF density, skin thickness, and HF depth, were augmented in rabbits with the patchiness phenotype relative to the non-patchiness phenotype. A total of 592 differentially expressed genes (DEGs) were identified between the two groups using RNA-sequencing. These included *KRT72*, *KRT82*, *KRT85*, *FUT8*, *SOX9*, and *WNT5B*. The functions of the DEGs were investigated by GO and KEGG enrichment analyses. A candidate gene, *KRT82*, was selected for further molecular function verification. There was a significant positive correlation between *KRT82* expression and HF-related parameters, and *KRT82* overexpression and knockdown experiments with rabbit dermal papilla cells (DPCs) showed that it regulated genes related to skin and HF growth and development. Investigation of single nucleotide polymorphisms (SNPs) in the exons and promoter region of *KRT82* identified four SNPs in the promoter region but none in the exons. The G.-631G > T, T.-696T > C, G.-770G > T and A.-873 A > C alleles conformed to the Hardy − Weinberg equilibrium, and three identified haplotypes showed linkage disequilibrium. Luciferase reporter assays showed that the core promoter region of *KRT82* was located in the − 600 to − 1200 segment, in which the four SNPs were located.

**Conclusions:**

The morphological characteristics of the patchiness phenotype were analyzed in New Zealand rabbits and DEGs associated with this phenotype were identified by RNA-sequencing. The biological functions of the gene *KRT82* associated with this phenotype were analyzed, and four SNPs were identified in the promoter region of the gene. These findings suggest that *KRT82* may be a potential biomarker for the breeding of experimental New Zealand rabbits.

**Supplementary Information:**

The online version contains supplementary material available at 10.1186/s12864-024-10370-7.

## Introduction

Rabbits are widely used as a source of meat, wool, and fur, as well as for scientific experiments [1]. Laboratory rabbits are bred in accordance with scientific rigor and ethical principles, and are usually used as models in the life sciences. New Zealand rabbits are one of the most commonly used rabbit breeds and are used as disease models, to study reproductive physiology, for antibody production, and for testing skin sensitization and irritation [2–5]. In vivo tests on rabbits serve as the benchmark for comparing skin irritation, for which there is no suitable alternative [5]. In skin sensitization and irritation tests, an experimental agent is usually applied to the shaved skin of the rabbit, and the skin response is scored according to established physiological parameters [6]. Many countries have framed relevant laws and regulations related to the use of rabbits in the cosmetic industry and for skin sensitization and irritation tests.

In general, skin sensitization and irritation tests are performed by shaving the dorsal skin of New Zealand rabbits. However, the dorsal skin in some New Zealand rabbits may show bulging, thickening, and irregular patches, all of which can interfere with precise evaluation. While the findings of various studies have provided evidence for potential regulatory mechanisms underlying different skin and hair follicle (HF) phenotypes in rabbits, there are no published reports describing the intrinsic regulatory mechanism. A previous study on the morphological characteristics of HFs in rabbit dorsal skin during the anagen phase showed that both the HF area and number of proliferating cell nuclear antigen (PCNA)-positive cells were greater in HFs in rabbits with thickened and erythematous skin relative to HFs from smooth skin [7]. Another study compared the skin histology of New Zealand rabbits and Angora rabbits, and found that the skin of the Angora rabbits was thicker, had more unit HFs in the dermis area, and had higher PCNA immunoreactivity than the skin of New Zealand rabbits [8]. In addition, differentially expressed genes (DEGs) associated with wool densities in the Rex rabbit were evaluated using gene expression microarrays, finding that *TGFβ1*, *GHR*, and *KAP6.1* regulated HF development [9]. Rex rabbits show characteristic wrinkles in the abdomen and extremities, also known as the plaice phenotype, which is regulated by the *LAMB3* gene [10].

In this study, the regulatory mechanism underlying irregular patches and skin thickening in New Zealand rabbits was investigated. DEGs related to HF and skin growth and development in the patchiness phenotype were screened and identified using RNA-sequencing. One of the key genes, *KRT82* (encoding keratin 82) was found to be highly expressed in the patchiness phenotype rabbit group. Previous studies have reported that this gene is expressed in the fiber cuticle, especially in the upper keratogenous zone, in sheep [11], while in Angora rabbits, *KRT82* was found to be differentially expressed between rabbits with coarse and fine wool [12]. Here, the molecular function of *KRT82* was investigated, and the polymorphisms and population genetic diversity associated with *KRT82* in the patchiness and non-patchiness groups were analyzed. This research provides a potential reference for the molecular breeding of New Zealand rabbit for experimental purposes.

## Results

### Morphological characteristics of the patchiness phenotype in New Zealand rabbits

**Fig. 1:**
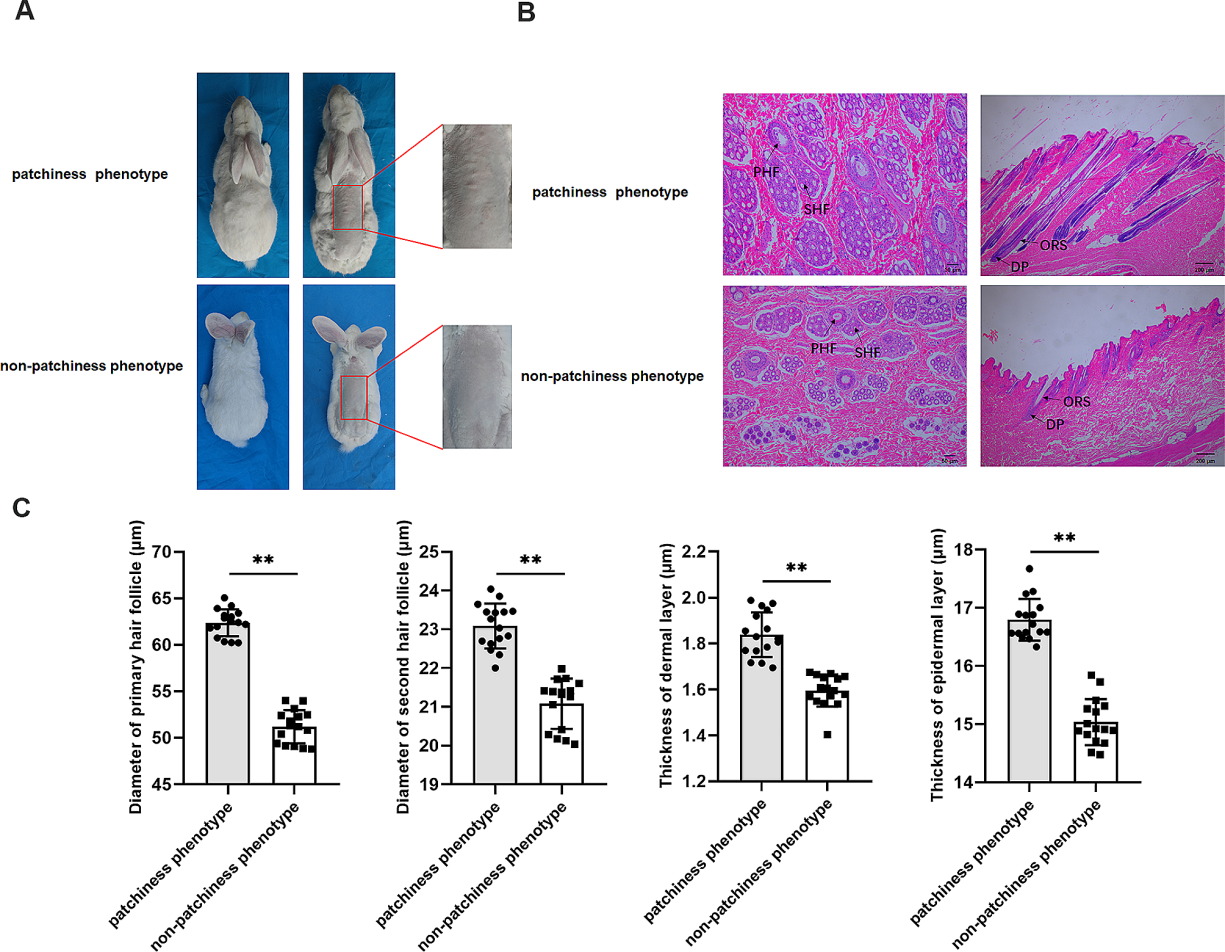
Morphological analysis of the dorsal skin between the patchiness and non-patchiness phenotypes in New Zealand rabbits. (**A**) Morphological observations of the patchiness phenotype in New Zealand rabbits. (**B**) Histology of transverse and longitudinal sections of the dorsal skin in rabbits with the patchiness and non-patchiness phenotypes. (**C**) HF-related parameters in the patchiness- and non-patchiness phenotype groups PHF: primary hair follicle, SHF: secondary hair follicle, DP: dermal papilla, ORS: outer root sheath. **P < 0.01

The morphology of the dorsal skin of the New Zealand rabbits is shown in Fig. 1A. The patchiness is difficult to recognize without shaving off the wool. After shaving the wool, the skin of rabbits with the non-patchiness phenotype appeared smooth with no swelling or protruberances, while that of rabbits with the patchiness phenotype showed irregular patches, thickening, and swelling on the dorsal skin. Histological sections of skin tissue from both groups were stained with HE (Fig. 1B). Transverse skin sections showed larger and greater numbers of primary HFs in the patchiness phenotype group relative to the non-patchiness group, while longitudinal sections indicated deeper and larger HFs in the patchiness group. The patchiness phenotype group had strong dermal papillae, significant outer root sheaths, and abundant extracellular matrix. Moreover, analysis of HF parameters (Fig. 1C) demonstrated higher densities of primary and secondary HFs, thicker dermal and epidermal layers, and larger diameters of primary and secondary HFs in the patchiness phenotype group compared with the non-patchiness group (*P* < 0.05).

### Screening of DEGs associated with the patchiness phenotype

**Fig. 2:**
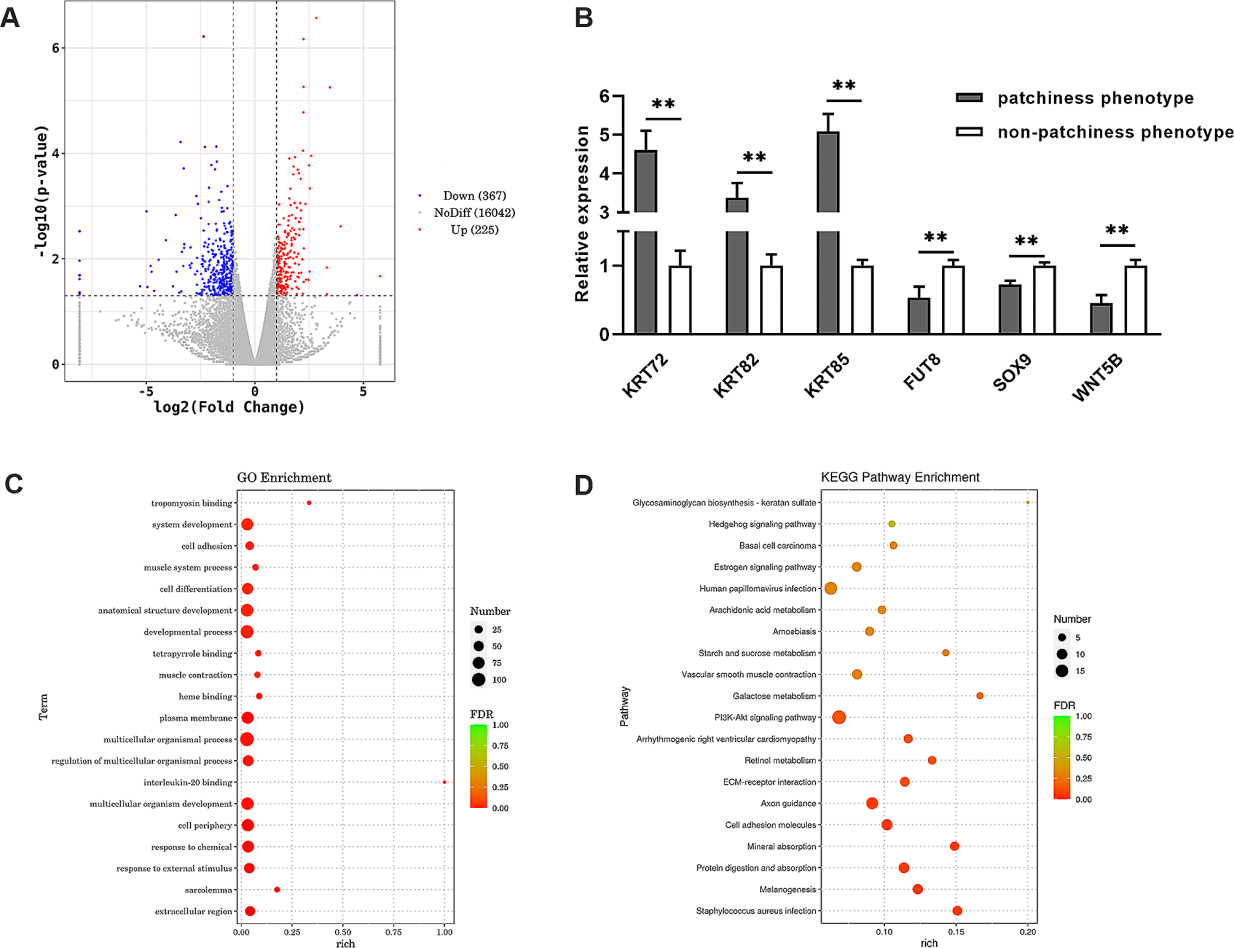
Identification of DEGs between the patchiness and non-patchiness phenotypes in New Zealand rabbits using RNA-sequencing. (**A**) Volcano plots showing upregulated and downregulated mRNAs between the patchiness and non-patchiness phenotypes. (**B**) Verification of differentially expressed genes between the patchiness and non-patchiness phenotypes. (**C**) GO enrichment analysis of DEGs between the patchiness and non-patchiness phenotypes. (**D**) KEGG enrichment analysis of DEGs between the patchiness and non-patchiness phenotypes **P < 0.01

Differentially expressed genes between the patchiness- and non-patchiness phenotypes were screened using RNA-sequencing. After quality evaluation and filtration of the raw sequencing data, the reads were mapped to the rabbit reference genome. The DEGs were identified according to their expression levels using the DESeq package in R with the criteria of |log_2_FoldChange|>1 and *P* < 0.05. The results identified 592 DEGs (225 upregulated and 367 downregulated), between the patchiness and non-patchiness phenotypes (Fig. 2A, Table S1). To confirm the expression levels of the DEGs, six genes (*KRT72*, *KRT82*, *KRT85*, *FUT8*, *SOX9*, and *WNT5B*) were selected for verification by RT-qPCR (Fig. 2B). The results showed that *KRT72*, *KRT82*, and *KRT85* were upregulated in the patchiness phenotype group while *FUT8*, *SOX9*, and *WNT5B* were downregulated, confirming the results of the DEG analysis. In addition, Gene Ontology (GO) and Kyoto Encyclopedia of Genes and Genomes (KEGG) were used to evaluate the functional enrichment of the DEGs (Fig. 2C and D). The genes were found to be enriched in various GO terms such as cell adhesion, cell differentiation, structural constituent of skin epidermis, receptor regulator activity and keratin filament, HF development, hair cycle, skin development, and skin epidermis development. The KEGG analysis showed enrichment in pathways associated with skin and HF development, including the Hedgehog signaling pathway, Wnt signaling pathway, Janus kinase (JAK)-signal transducer and activator of transcription (STAT) signaling pathway, and mitogen-activated protein kinase (MAPK) signaling pathway.

### Pearson correlation analysis of ***KRT82*** gene expression and HF-related parameters

**Fig. 3:**
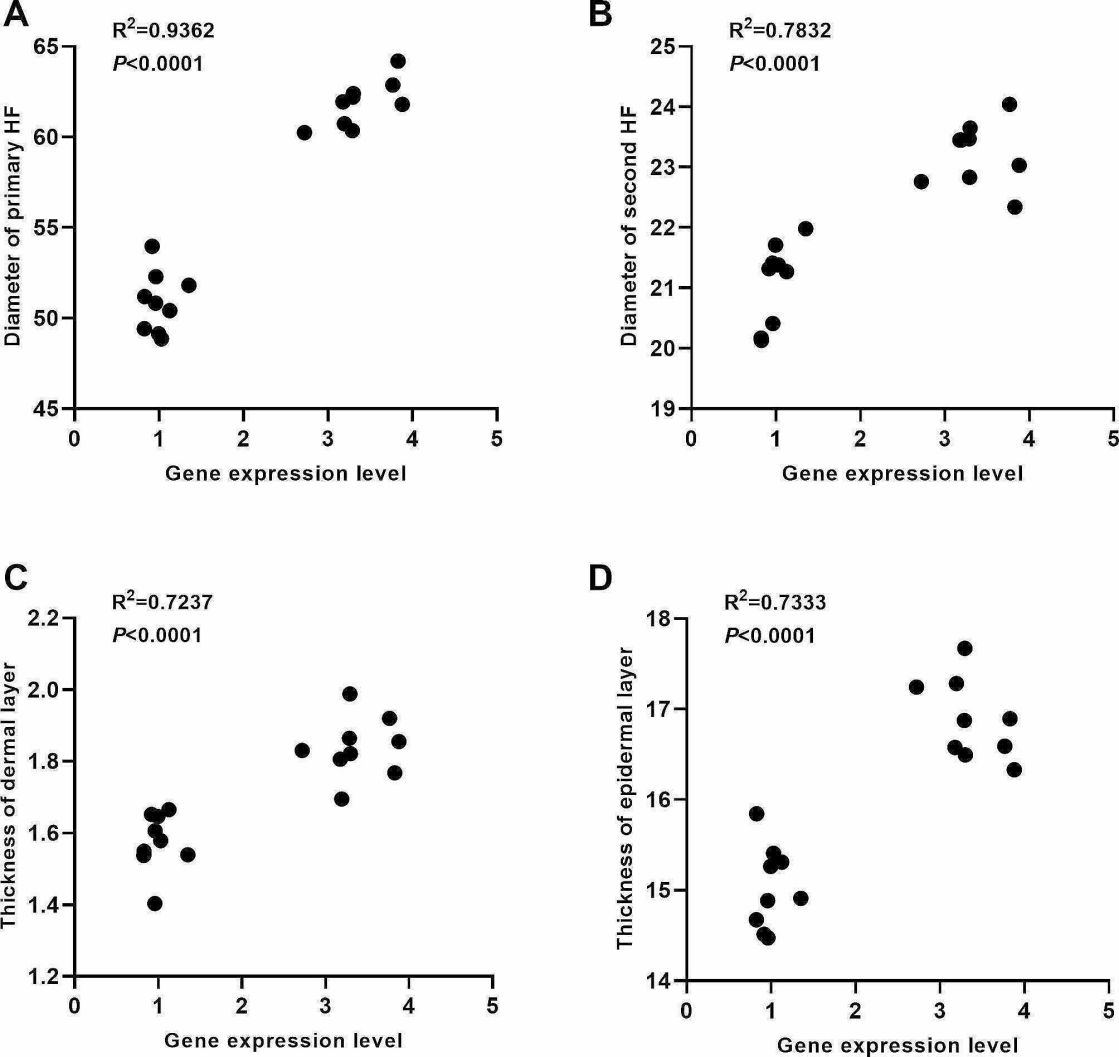
Pearson correlation analysis of *KRT82* gene expression levels and HF-related parameters. (**A**) Correlations between *KRT82* expression and primary HF diameter. (**B**) Correlations between *KRT82* expression and secondary HF diameter. (**C**) Correlations between *KRT82* gene expression and dermal layer thickness. (**D**) Correlations between *KRT82* expression and epidermal layer thickness

As shown by the DEG analysis, *KRT82* expression was significantly upregulated in the patchiness phenotype group, and functional enrichment showed that *KRT82* was a structural constituent of skin epidermis, keratinization, and keratin filaments. Thus, it was speculated that *KRT82* was a candidate gene that could regulate the patchiness phenotype in New Zealand rabbits. To determine the association of HF-related parameters with the patchiness phenotype, Pearson correlation analysis was performed, showing correlation coefficients R^2^ of 0.9362 (*P* < 0.0001), 0.7832 (*P* < 0.0001), 0.7237 (*P* < 0.0001), and 0.7333 (*P* < 0.0001) between the primary HF diameter, secondary HF diameter, dermal layer thickness, epidermal layer thickness, and *KRT82* mRNA expression, respectively (Fig. 3). Thus, there were significant positive correlations between *KRT82* expression and HF-related parameters.

### Cloning and bioinformatics analysis of KRT82

The coding sequence of *KRT82* was cloned, showing that the 1524-bp open-reading frame (ORF) sequence encoded 507 amino acids. Bioinformatics analysis showed that the molecular formula of KRT82 was C_2438_H_3972_N_698_O_772_S_26_, with a molecular weight of 56 248.13 Da, a theoretical pI of 7.98, an instability index (II) of 53.68, and a grand average of hydropathicity (GRAVY) value of − 0.545, indicating that KRT82 is an unstable hydrophilic protein. Predictions by SignalP and TMHMM indicated that KRT82 did not have putative signal peptide nor transmembrane domains, respectively. Protein structure prediction showed that the secondary structure of KRT82 contained 59.57% α-helix, 31.76% random coil, 7.69% extended strand, and 0.99% β-turn. Its tertiary structure comprised a helical chain, as predicted by SWISS-MODEL. The PPI network constructed by the STRING database showed that KRT31, KRT27, KRT35, and KRT39 were interacted with KRT82 (Figure S1).

### Regulation of KRT82 overexpression and knockdown in DPCs

**Fig. 4:**
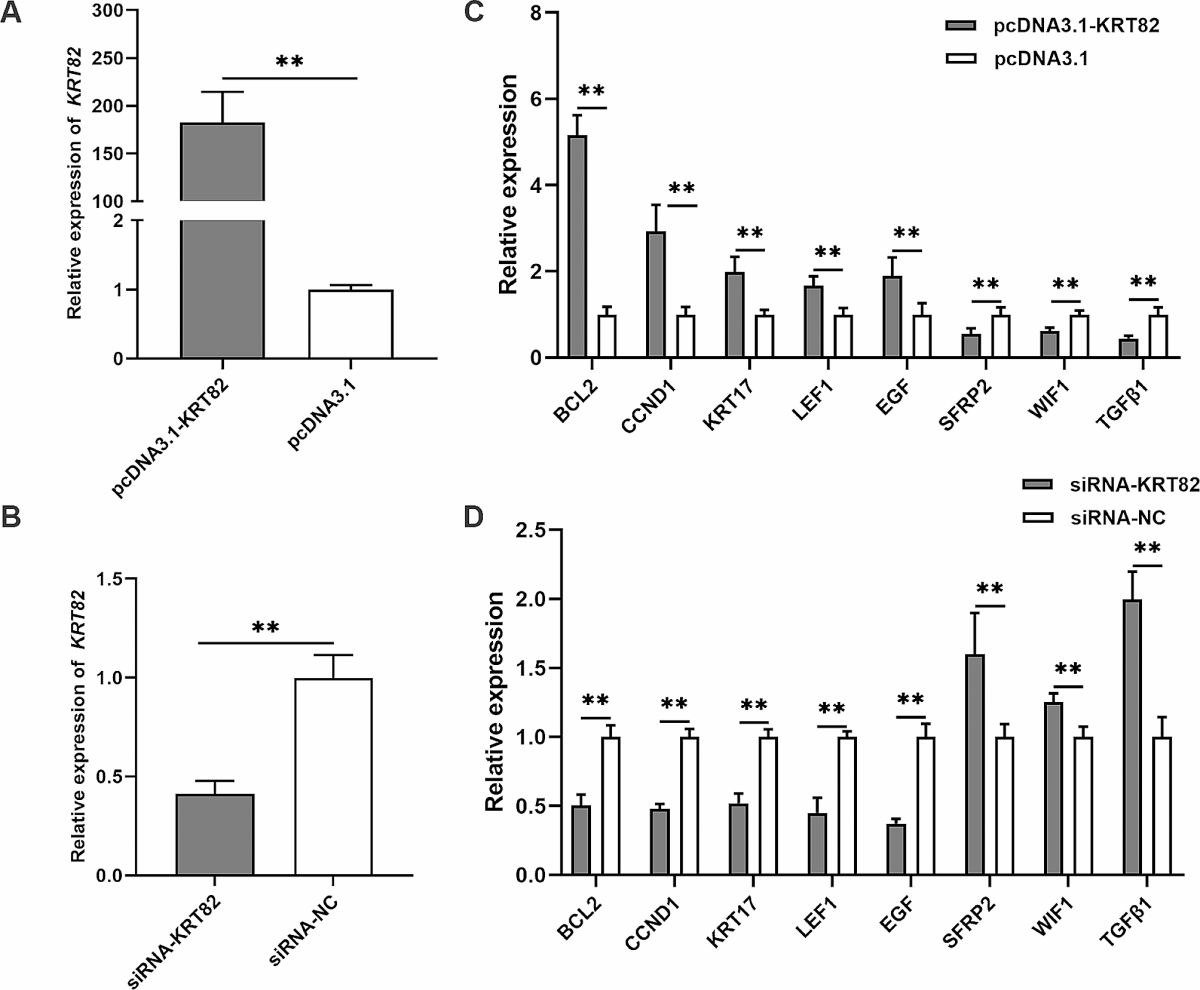
Overexpression and knockdown of *KRT82* regulates HF growth- and development-related genes. (**A**) Overexpression of *KRT82* in rabbit DPCs. (**B**) Knockdown of *KRT82* in rabbit DPCs. (**C**) Effects of *KRT82* overexpression on HF development-related genes. (**D**) Effects of *KRT82* knockdown on HF development-related genes **P < 0.01

An overexpression vector, pcDNA3.1-KRT82, was then constructed and siRNAs were designed to explore the gene expression of *KRT82*. RT-qPCR results showed that pcDNA3.1-KRT82 significantly upregulated *KRT82* mRNA expression (*P* < 0.01, Fig. 4A) and that siRNA-2 could significantly downregulate *KRT82* mRNA expression (*P* < 0.01, Fig. 4B) in DPCs. *KRT82* overexpression significantly downregulated the mRNA expression of *SFRP2*, *TGFβ1*, and *WIF1* (*P* < 0.01), but upregulated that of *BCL2*, *CCND1*, *EGF*, *LEF1*, and *CTNNB1* (*P* < 0.01, Fig. 4C). Further, the knockdown of *KRT82* led to upregulation of *SFRP2*, *TGFβ1*, and *WIF1* (*P* < 0.01), as well as downregulation of *BCL2*, *CCND1*, *EGF*, *LEF1*, and *CTNNB1* (*P* < 0.01, Fig. 4D).

### SNP detection and population genetic diversity analysis of ***KRT82***

**Fig. 5:**
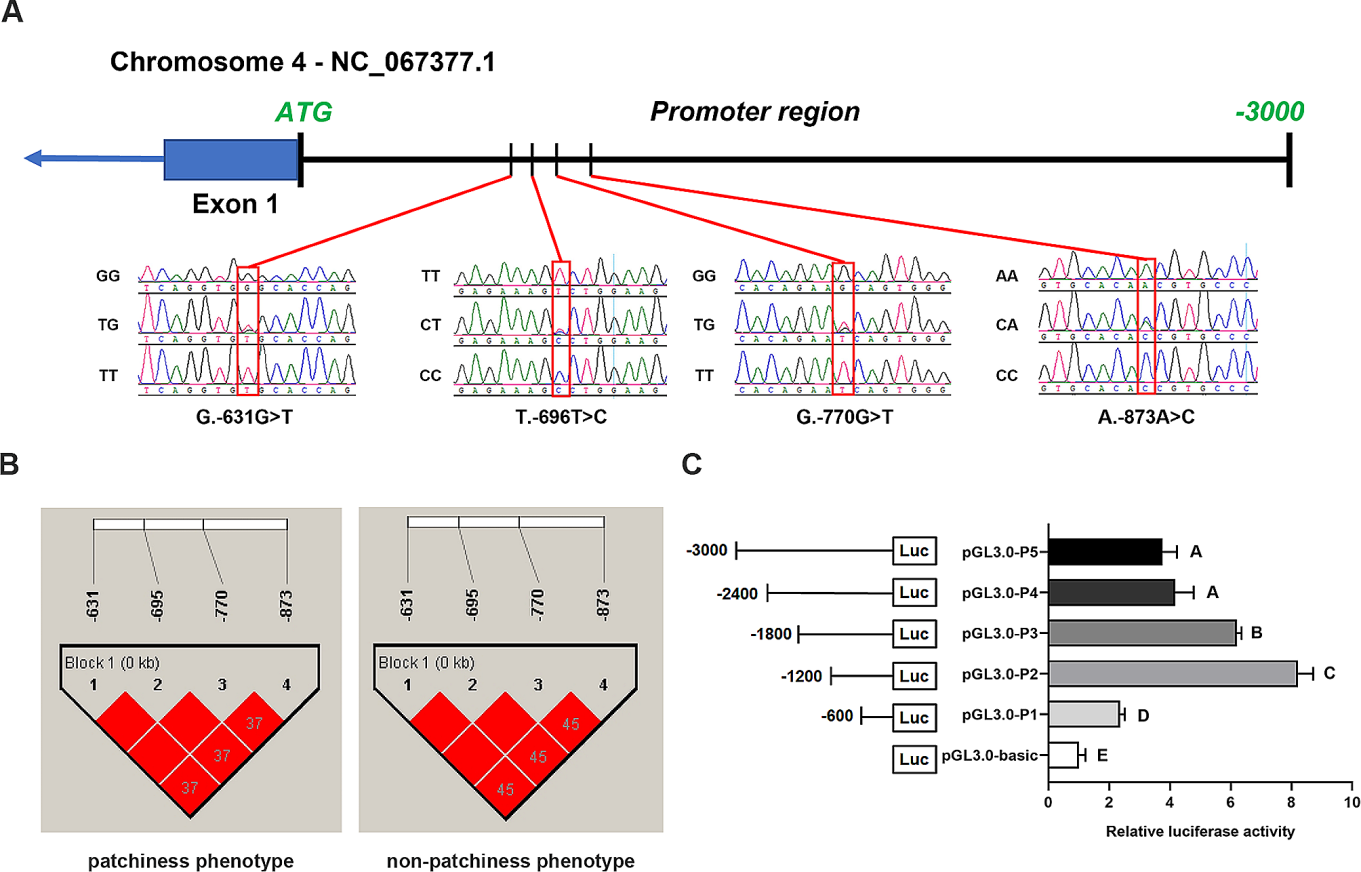
Single-nucleotide polymorphisms in the promoter region of *KRT82* in the patchiness and non-patchiness phenotypes. (**A**) Distribution of single-nucleotide polymorphisms in the promoter region of *KRT82*. (**B**) Linkage disequilibrium analysis of the *KRT82* single-nucleotide polymorphisms. (C) Luciferase activity in the *KRT82* promoter region Different letters indicate significant differences (*P* < 0.05)

The presence of SNPs in the nine exons of *KRT82* was investigated between patchiness phenotype (*n* = 50) and non-patchiness phenotype (*n* = 50) rabbits using PCR amplification and direct sequencing. However, no SNPs were found in these exons. We also investigated the presence of SNPs in the 3000 nt upstream of ATG in *KRT82* between the two groups. The results revealed four SNPs located at the − 631 (G > T), − 696 (T > G), − 770 (G > T), and − 873 (A > C) loci upstream of the ATG of *KRT82* (Fig. 5A). The genotypic frequency, allele frequency, and population genetic diversity were analyzed (Table 1). In both the patchiness and non-patchiness phenotype groups, G.-631G > T, T.-696T > C, G.-770G > T, and A.-873 A > C showed moderate polymorphism (0.25 < PIC < 0.5). Chi-square tests (χ^2^ tests) showed that G.-631G > T, T.-696T > C, G.-770G > T, and A.-873 A > C conformed to the Hardy − Weinberg equilibrium (HWE, *P* > 0.05), with low heterozygosity (He) and high homozygosity (Ho).

**Table 1:** Genotype distribution and allele frequencies of the SNPs in the promoter region of *KRT82*

Group	SNPs	genotype frequency			allele frequency		He	Ho	c^2^	PIC	Hardy–Weinberg *P*-Value
patchiness phenotype (*n* = 50)	G.-631G > T	GG	GT	TT	G	T	0.3318	0.6682	0.4593	0.2768	0.8002
		0.64	0.30	0.06	0.79	0.21					
	T.-696T > C	TT	TC	CC	T	C	0.3318	0.6682	0.4593	0.2768	0.8002
		0.64	0.30	0.06	0.79	0.21					
	G.-770G > T	GG	GT	TT	G	T	0.3318	0.6682	0.4593	0.2768	0.8002
		0.64	0.30	0.06	0.79	0.21					
	-873 A > C	AA	AC	CC	A	C	0.4662	0.5338	1.2530	0.3575	0.5363
		0.36	0.54	0.10	0.63	0.37					
non-patchiness phenotype (*n* = 50)	G.-631G > T	GG	GT	TT	G	T	0.4488	0.5512	1.9574	0.3481	0.3753
		0.48	0.36	0.16	0.66	0.34					
	T.-696T > C	TT	TC	CC	T	C	0.4488	0.5512	1.9574	0.3481	0.3753
		0.48	0.36	0.16	0.66	0.34					
	G.-770G > T	GG	GT	TT	G	T	0.4488	0.5512	1.9574	0.3481	0.3753
		0.48	0.36	0.16	0.66	0.34					
	-873 A > C	AA	AC	CC	A	C	0.4872	0.5128	1.1164	0.3685	0.5732
		0.14	0.56	0.30	0.42	0.58					

### Linkage disequilibrium analysis of ***KRT82***

The linkage disequilibrium (LD) and haplotype of the SNPs were analyzed by Haploview 4.2 software. As shown in Fig. 5B, a haplotype block was constructed at the four SNPs in the patchiness phenotype and non-patchiness phenotype rabbits, and the four SNPs in the two groups were found to be fully linked (R^2^ = 1). In the patchiness phenotype group, there were three haplotypes, including H1 (GTGA), H2 (TCTC), and H3 (GTGC) with frequencies of 0.4200, 0.3400, and 0.2400, respectively. In the non-patchiness phenotype group, the frequency of the three haplotypes (H1, H2, and H3) was 0.6300, 0.2100, and 0.1600, respectively (Table 2). Based on the pairwise combination of the three haplotypes, five combinations of diplotypes were found in the two groups, of which the frequencies of H2H2 and H2H3 in the non-patchiness phenotype group were lower than 0.07 (Table 3).

**Table 2:** Haplotypes and frequencies of *KRT82* in rabbits with the patchiness and non-patchiness phenotypes

Group	Haplotype	SNP1	SNP2	SNP3	SNP4	Frequency
patchiness phenotype	H1	G	T	G	A	0.4200
	H2	T	C	T	C	0.3400
	H3	G	T	G	C	0.2400
non-patchiness phenotype	H1	G	T	G	A	0.6300
	H2	T	C	T	C	0.2100
	H3	G	T	G	C	0.1600

**Table 3:** Diplotypes and frequencies of *KRT82* in rabbits with the patchiness and non-patchiness phenotypes

Group	Diplotypes	SNP1	SNP2	SNP3	SNP4	Frequency
patchiness phenotype	H1H1	GG	TT	GG	AA	0.1400
	H1H2	GT	TC	GT	AC	0.2200
	H1H3	GG	TT	GG	AC	0.3400
	H2H2	TT	CC	TT	CC	0.1600
	H2H3	GT	TC	GT	CC	0.1400
non-patchiness phenotype	H1H1	GG	TT	GG	AA	0.3600
	H1H2	GT	TC	GT	AC	0.2600
	H1H3	GG	TT	GG	AC	0.2800
	H2H2	TT	CC	TT	CC	0.0600
	H2H3	GT	TC	GT	CC	0.0400

### Analysis of ***KRT82*** promoter activity

The promoter activity of *KRT82* was examined using luciferase reporter assays. The results showed that the − 600 to − 1200 segment of the *KRT82* promoter region had the highest luciferase activity (Fig. 5C). Interestingly, the SNPs G.-631G > T, T.-696T > C, G.-770G > T, and A.-873 A > C were located in this core promoter region. Using the animal TFDB prediction software, we found that the polymorphism in promoter region caused changes in transcription factor (TF) binding sites (Table S6). In summary, the SNP G.-631G > T results in the gain of fifteen TFs binding sites (e.g. *MGA*, *MTF1* and *PAX6*), and the loss of eleven TFs binding sites (e.g. *FIGLA*, *HIC1* and *HIC2*). The T.-696T > C caused the gain of three TFs binding sites, *Blimp-1*, *Hnf4a*, *Rela*, and the loss of seventeen binding sites (e.g. *ELK4*, *HKR1*, *SP1* and *SP5*). In addition, the mutant of G.-770G > T results in the loss of twenty-two specific TFs binding sites (e.g. *KLF5*, *MAZ*, *OSR1* and *SP5*). The SNP A.-873 A > C caused the gain of twelve TFs binding sites (e.g. *ESR1*, *KLF3*, *MTF1* and *PAX6*), and loss of seven TFs binding sites (e.g. *ARNT*, *EPAS1* and *HIF1A*), respevtively. However, further investigation is required to explore the associations between transcription-factor binding and polymorphisms in the *KRT82* promoter region.

## Discussion

Rabbits are used as experimental animals to test sensitivities to different chemical compounds [13]. In comparison with human skin, rabbit skin is more sensitive, which can help researchers to accurately predict whether a substance causes skin irritation [14]. Furthermore, the permeability of the rabbit skin is higher than that of rats, pigs, and humans [15]. Morphological analysis revealed the presence of irregular patches on the dorsal skin of the rabbits, together with deeper HFs, higher HF densities, and greater HF diameters, with the patchiness phenotype compared with those with the non-patchiness phenotype. Alopecia areata (AA) is a condition leading to bald spots on the scalp area or, in some cases, loss of all the hair from the scalp or the body [16]. AA-like diseases have also been reported in non-human mammalian species [17] such as the laboratory rat [18], mouse [19], dog [20], horse [21], cow [22] and non-human primates [23]. No research has been published on the mechanism of AA in rabbits. Rabbits with irregular patches and complete HF structures but without obvious pathology suggest the presence of a different mechanism responsible for the irregular patches on the dorsal skin from that seen in AA.

In a previous study, DEGs associated with skin and HF development were identified using RNA-sequencing. For example, Rex rabbits exhibit a wrinkle phenotype, and DEGs associated with the development of wrinkled skin were identified by RNA-sequencing [10]. The DEGs between the back and belly skin in the Chinchilla Rex rabbit were found to be involved in fur development [24]. DEGs have also been identified between short-haired and long-haired rabbits, and were found to participate in pathways involved in hair growth [25]. To explore the potential regulatory mechanism of the patchiness phenotype, we examined DEGs between rabbits with the patchiness phenotype and those lacking the phenotype, identifying *KRT39*, *KRT82*, *KRT85*, *LEF1*, *WNT5A*, and *MSX2* as candidate genes. Various skin- and HF-related signaling pathways, including the Hedgehog, Wnt, JAK-STAT, and MAPK signaling pathways, could be involved in the regulation of structural changes in rabbit skin and HFs.

The RNA-sequencing results showed that the expression of *KRT39*, *KRT72*, *KRT82*, and *KRT85* was upregulated in the patchiness phenotype group, indicating the involvement of keratin proteins in the development of the patchiness phenotype. Keratin proteins (KRTs) represent the principal structural components of skin, hair, and wool, and regulate their growth and development [26]. *KRT39* was found to be differentially expressed between the fine- and coarse-type skin tissues in cashmere goats and to regulate the growth of wool fibers [27]. *KRT82* was also shown to be expressed in the hair shaft cuticle during HF anagen, and its expression was downregulated in the skin and HFs of patients with AA [28]. In the wool follicle, *KRT85* was detected in the primary follicle during anagen and was strongly expressed in the cortex on the inner side of the hair bulb in curved secondary follicles [29]. Wool fibers are composed mainly of keratin proteins, and *KRT85* was found to be expressed in the lower bulb, while *KRT82* was expressed in the fiber cuticle [11]. *KRT82* was also found by RNA-sequencing to be differentially expressed between coarse and fine wool in the Angora rabbit [12]. In mouse skin, *KRT82* is differentially expressed in interfollicular keratinocytes, HFs, and dermal fibroblasts, including dermal papilla cells, as shown by RNA-sequencing [30]. Furthermore, the present study found a significant positive correlation between HF-related parameters and *KRT82* mRNA expression, suggesting that *KRT82* acts as a key regulator of the development of irregular patches, thickening, and swelling on the dorsal skin of New Zealand rabbits. Bioinformatics analysis predicted that rabbit KRT82 is an unstable and hydrophilic protein and its overexpression or knockdown in DPCs could have important consequences on the expression of genes associated with growth and development in the skin and HF, such as *SFRP2* [31], *TGFβ1* [32], *WIF1* [33], *BCL2* [34], *CCND1* [35], *LEF1* [36], *CTNNB1* [37], and *EGF* [38], indicating that *KRT82* plays an important role in the regulation of skin and HF development.

Many SNPs have been found to be associated with skin and HF development in rabbits. For instance, in the Rex rabbit, SNPs identified in *CCNA2* were found to be associated with wool density [39]. The key SNP in the promoter region of the *WIF1* gene was thought to be related to the length of wool in the rabbit [40]. A nonsynonymous nucleotide substitution in *FGF5* in Rex, New Zealand, and Angora rabbits was associated with the long-hair trait [41]. In this study, no SNPs were found in the nine exons of *KRT82* that differed between rabbits with the patchiness phenotype and control rabbits, indicating that the *KRT82* gene is strongly conserved in different New Zealand rabbits with different dorsal-skin phenotypes. However, we identified four SNPs in the promoter region of *KRT82* where the G.-631G > T, T.-696T > C, G.-770G > T, and A.-873 A > C polymorphisms conformed to the HWE and were located in the core promoter region. Polymorphisms in the promoter regions of genes play important roles in the expression of those genes and thus regulation of the phenotype [42–44]. Changes in transcription factor-binding sites in the promoter regions can cause significant dysregulation of the transcription and thus expression of genes [45]. However, the relationships between transcription factors and the SNPs identified in the *KRT82* promoter region should be investigated further. Future work should address the mechanism underlying the patchiness phenotype in rabbits using genome selection to provide a reference for the selective breeding of New Zealand rabbits lacking this phenotype.

## Conclusion

In the current study, the morphological characteristics of the patchiness phenotype in New Zealand rabbits were analyzed, and the DEGs between the patchiness and non-patchiness phenotypes were identified using RNA-sequencing. The biological functions of the candidate gene *KRT82* were investigated. *KRT82* was found to regulate genes involved in the growth and development of the skin and HFs. No SNPs were found in the exons, although four SNPs were identified in the promoter region of *KRT82* that were found to be associated with the patchiness phenotype in New Zealand rabbits. These findings indicate the involvement of *KRT82* in the regulation of the patchiness phenotype in New Zealand rabbits and its potential role as a novel biomarker for the selective breeding of New Zealand rabbits for experimental use.

## Materials and methods

### Experimental animals and sample collection

Six-month-old New Zealand white rabbits were obtained from Jiangsu Province Dongfang Rabbit Co., Ltd. (China). The animals were housed in a temperature- and humidity-controlled environment, and were fed the same diet. For sample collection, the rabbits were anesthetized by intravenous injection of Zoteil-50 into the ear vein, followed by the application of an iodine solution to the wound to avoid bacterial infection and continue feeding. The dorsal skin (1 cm^2^) was collected for RNA extraction, and an ear sample (1 cm^2^) was obtained for DNA extraction. Dorsal skin samples were fixed in 4% formaldehyde, and paraffin sections were stained with hematoxylin–eosin (HE) for histological evaluation.

### Cell culture and transfection

Dermal papilla cells (DPCs) were separated from the rabbit HF and cultured in mesenchymal stem cell medium (ScienCell). The RAB-9 cell line (CRL1414™) was purchased from the American Type Culture Collection (ATCC) and maintained in Minimum Essential Medium (MEM, Gibco) supplemented with 10% fetal bovine serum (FBS, One Shot™, Gibco). The cells were maintained and cultured in a humidified incubator in the presence of 5% carbon dioxide (CO_2_) at 37℃. For cell transfection, Lipofectamine™ 2000 (Invitrogen, MA, USA) was used according to the manufacturer’s instructions.

### RNA isolation and real-time quantitative polymerase chain reaction (RT-qPCR)

Total RNA was extracted from the rabbit skin or cells using the RNAsimple Total RNA Kit (Tiangen, Beijing, China). The cDNA was obtained using HiScript II Q Select RT SuperMix (Vazyme, Nanjing, China) and treated with the AceQ qPCR SYBR® Green Master Mix (Vazyme) for determination of the relative expression levels of genes on a QuantStudio® 5 system (Applied Biosystems, Thermo Fisher Scientific, Waltham, MA, USA). The rabbit glyceraldehyde 3-phosphate dehydrogenase (*GAPDH*) gene served as the reference, and relative gene expression was estimated by the 2^−ΔΔCt^ method [46]. Primer sequences are listed in Table S2.

### RNA library construction and RNA-sequencing

Samples from rabbits with the patchiness and non-patchiness phenotypes were processed for RNA sequencing on an Illumina HiSeq 2500 high-throughput sequencing platform. The dorsal skin from rabbits with the patchiness phenotype (*n* = 3) and non-patchiness phenotype (*n* = 3) rabbits were collected to prepare RNA. The concentration and purity of the total RNA were quantified using a Qubit 3.0 fluorometer (Invitrogen, Waltham, MA, USA) and an Agilent 2100 bioanalyzer (Applied Biosystems, Carlsbad, CA, USA), respectively. Libraries were constructed and library quality was determined using the Agilent 2100 bioanalyzer (Applied Biosystems, Waltham, MA, USA). Paired-end clean reads were aligned using HISAT2 to the reference genome of *Oryctolagus cuniculus* (OryCun2.0) obtained from Ensembl. The mapped mRNA reads from each sample were then assembled using StringTie, and the fragments per kilo-base millions of exons per million fragments mapped (FPKM) of the mRNA in each sample were calculated. The differentially expressed mRNAs between patchiness and non-patchiness phenotype groups were determined using the DESeq package in R, and DEGs were identified using the criteria of |log2FoldChange|>1 and *P* < 0.05. To better understand their biological functions, the enrichment of the DEGs in GO and KEGG pathways was analyzed. GO analysis classified the DEGs into three categories, namely, molecular function (MF), biological process (BP), and cellular component (CC), and KEGG pathway analysis was performed to predict DEG-related pathways.

### Overexpression and knockdown of ***KRT82***

For the cloning of the rabbit *KRT82* gene, total RNA was obtained from the rabbit skin using RNAsimple Total RNA Kit (Tiangen, Beijing, China), and cDNA was synthesized using the PrimeScript™ 1st Strand cDNA Synthesis Kit (Takara, Dalian, China). According to the rabbit *KRT82* mRNA sequence (GenBank accession no. XM_051845525.1), an overexpression vector of KRT82 was constructed, and the CDS sequence of *KRT82* was subcloned into a *HindIII*- and *EcoRI*-digested pcDNA3.1(+) vector. For the knockdown of *KRT82* expression, small-interfering RNA (siRNA) and siRNA-NC were designed and purchased from Shanghai GenePharma Co., Lt. Primers used for construction of the overexpression vector and siRNA sequences are shown in Table S3.

### Bioinformatic analysis of KRT82

The KRT82-coding sequence was analyzed by the DNASTAR software package (DNASTAR). Using the online software ProtParam (http://web.expasy.org/protparam/) [47], the molecular weight, molecular formula, instability coefficient, and isoelectric point (pI) of the KRT82 protein were predicted. The signal peptide, localization signal, protein transmembrane region, and secretory unit of KRT82 were predicted using SignalP 4.1 (http://www.cbs.dtu.dk/services/SignalP-4.1/) [48] and TMHMM 2.0 (https://services.healthtech.dtu.dk/service.php?TMHMM-2.0) [49]. The secondary structure and three-dimensional homology model of the KRT82 protein were predicted by Hopfield (http://npsa-pbil.ibcp.fr/cgi-bin/npsa_automat.pl?page=npsa_gor4.html) [50] and SWISS-MODEL [51], respectively. The STRING database was used to construct the protein-protein interaction (PPI) network of KRT82 [52].

### Identification of ***KRT82*** exon and promoter polymorphisms

The genomic DNA from rabbit ear samples in the patchiness phenotype group (*n* = 50) and non-patchiness phenotype group (*n* = 50) was extracted using the TIANamp Genomic DNA Kit (Tiangen), and the purity, concentration, and integrity of the DNA samples were analyzed by ultramicro spectrophotometry and 1% agarose gel electrophoresis. Samples with satisfactory purity and concentration and intact and bright bands were stored at − 20 °C and selected for subsequent experiments. Nine pairs of primers for the *KRT82* exons and five pairs of primers for the *KRT82* promoter region 3000 bp upstream of ATG were designed using the National Center for Biotechnology Information (NCBI) Primer BLAST software (Table S4). DNA samples were subjected to PCR amplification using 2× Rapid Taq Master Mix (Vazyme, Nanjing, China) according to the manufacturer’s instructions. The PCR products were then sequenced using Sanger sequencing.

### Dual-luciferase assay for ***KRT82*** promoter region verification

For the verification of its core promoter region, *KRT82* promoter region segments were cloned into pGL3-Basic vectors; the primers are listed in Table S5. The luciferase reporter gene vectors were transfected into RAB-9 cells, and the luciferase activity was detected using a dual-luciferase reporter system (Promega, Madison, WI, USA). *Renilla* luciferase activity was used for the normalization of firefly luciferase activity. The TF of the promoter region sequence was predicted using the online tool AnimalTFDB v4.0 (https://guolab.wchscu.cn/AnimalTFDB4//#/) [53].

### Statistical analysis

Statistical analysis was conducted using SPSS 25.0 (IBM Corp., Armonk, NY, USA). The mRNA relative expression, luciferase activity, and HF-related parameters were analyzed using a two-tailed Student’s *t*-test or one-way analysis of variance (ANOVA). For the analysis of agreement with the Hardy-Weinberg equilibrium, allele frequencies of each SNP were calculated using the chi-square test (χ2-test). The correlation coefficients between HF-related parameters and *KRT82* gene expression were analyzed by Pearson correlation analysis. In addition, allele frequency, genotype distribution, heterozygosity, homozygosity, and polymorphism information content (PIC) were calculated by Microsoft Excel, while the haplotype analysis was carried out with Haploview 4.2 software. All error bars represent mean ± standard deviation (SD), and each analysis contained at least three biological replicates. Graphs were generated using GraphPad Prism 8 software (GraphPad Software, Inc., San Diego, CA, USA).

### Electronic supplementary material

Below is the link to the electronic supplementary material.

Supplementary Material 1

Supplementary Material 2

Supplementary Material 3

Supplementary Material 4

Supplementary Material 5

Supplementary Material 6

Supplementary Material 7

## Data Availability

The data was presented in the manuscript and the supporting materials. The raw sequencing data were deposited in the NCBI Short Read Archive (SRA) under BioProject accession number PRJNA1012882 (https://www.ncbi.nlm.nih.gov/bioproject/PRJNA1012882/).
