# Supplementary figures and images for: Single nucleotide polymorphisms in the KRT82 promoter region modulate irregular thickening and patchiness in the dorsal skin of New Zealand rabbits
Source: BMC Genomics. 2024 May 10;25:458. doi: 10.1186/s12864-024-10370-7 (PMC11088042; doi:10.1186/s12864-024-10370-7)

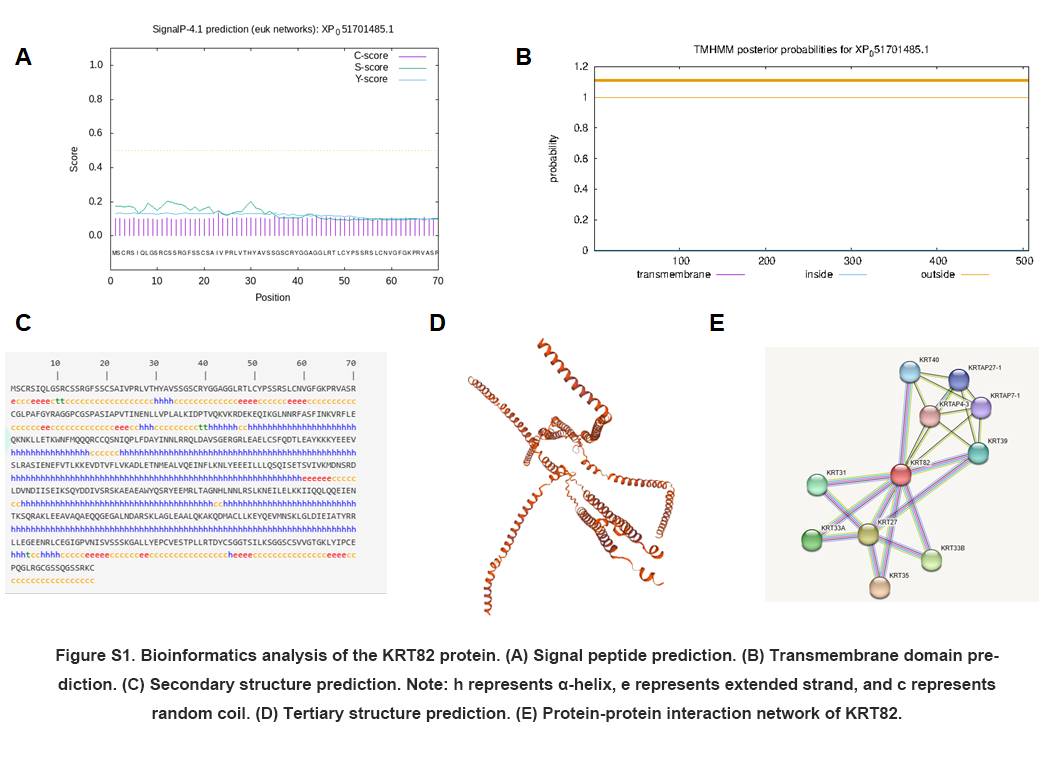

Supplement: Supplementary file 7 — Supplementary Material 7 [file 12864_2024_10370_MOESM7_ESM.png]
